# Clinical and Radiological Features of Urachal Carcinoma and Infection

**DOI:** 10.3389/fonc.2021.702116

**Published:** 2021-09-07

**Authors:** Shichao Li, Xiaoyan Meng, Ping Liang, Cui Feng, Yaqi Shen, Daoyu Hu, Zhen Li

**Affiliations:** Tongji Hospital, Tongji Medical College, Huazhong University of Science and Technology, Wuhan, China

**Keywords:** urachal carcinoma, urachal infection, radiological features, differential diagnosis, urachus

## Abstract

**Purpose:**

To explore the clinical and radiological differences between urachal carcinoma and urachal infection.

**Methods:**

Clinical and imaging information for 13 cases of urachal carcinoma and 14 cases of urachal infection confirmed by pathology were retrospectively analyzed. The size, location, shape, margin, lesion composition, calcification, T1 and T2 signal intensity, peripheral lymph nodes, degree of enhancement, adjacent bladder wall, and apparent diffusion coefficient (ADC) value were examined in both groups, and distinguish features were determined. The student *t*-test or Mann-Whitney *U* test was used for quantitative data, and Fisher’s exact test was used for qualitative data. Kappa coefficient consistency test was used to evaluate the interobserver agreement.

**Results:**

Sex, hematuria, abdominal pain, calcification, and thickening of adjacent bladder wall can distinguish between urachal carcinoma and urachal infection (*p* < 0.05). There were no statistical differences in age (*p* = 0.076), size (*p* = 0.797), location (*p* = 0.440), shape (*p* = 0.449), margin (*p* = 0.449), lesion composition (*p* = 0.459), T1 signal intensity (*p* = 0.196), T2 signal intensity (*p* = 0.555), peripheral lymph nodes (*p* = 0.236), degree of enhancements (*p* = 0.184) and ADC value (*p* = 0.780) between two groups.

**Conclusion:**

The following clinical and imaging features help distinguish urachal carcinoma from urachal infection: sex, hematuria, abdominal pain, calcification, and thickening of the adjacent bladder wall.

## Introduction

Urachus is a tubular structure extending from the front dome of the bladder to the umbilicus (1). During normal development, it eventually disappears and forms the median umbilical ligament. Approximately one-third of the human urachus may not be completely occluded before birth and infancy, and still exist in adulthood (2, 3). These embryological abnormalities include patent urachus, urachal cyst, urachal sinus, and vesicourachal diverticulum (4). Although usually asymptomatic, these remnants may cause infection, inflammation, fistulae, umbilical discharge, abdominal pain, or even, malignant transformation in the following years (5, 6).

Both tumors and infections are common complications of adult urachal abnormalities. Urachal carcinoma is a rare and highly malignant tumor with low morbidity, accounting for only 0.01% of all cancers and 0.34% of all bladder neoplasms annually, and it mainly occurs in men (7–10). Most urachal cancers are adenocarcinomas, including mucinous gland type, intestinal type, mixed type, signet ring cell type, and so on (11, 12). Other pathological types include transitional cell carcinoma, squamous cell carcinoma, small cell carcinoma, and neuroendocrine carcinoma, etc. (11, 13). Painless gross hematuria is the most common symptom, and when it occurs, it usually indicates that the tumor has already invaded the bladder (14). As for urachal infection, it is more common in infants and children (15) and may produce non-specific symptoms such as abdominal pain, fever, umbilical discharge, and a palpable abdominal mass (16).

According to literature and routine clinical practice, urachal carcinoma may manifest similar clinical symptoms to the infected urachal remnants, such as abdominal pain, hematuria, dysuria, etc., or both may be asymptomatic (15, 16). Kim et al. (17) reported that the urachal carcinoma may contain cystic components, and the tumors range from solid to the mixture of solid and cystic. In clinical practice, the composition of urachal infection lesions lacks specificity and can be manifested as cystic, solid, and mixed cystic and solid lesions, similar to tumor manifestations. Dynamic contrast-enhanced scanning is often used to distinguish tumors from other diseases, but both urachal carcinoma and infection could show varying degrees of enhancement, and in different ways. And both may demonstrate restricted diffusion on diffusion-weighted images. In the study of Carolina Parada Villavicencio et al., it was also concluded that the infection of the urachal remnants may mimic urachal carcinoma, resulting in challenges for imaging diagnosis (16). However, the treatment and prognosis between these two diseases are essentially different, and effective preoperative identification is of great significance. Antibiotics and complete resection of the urachal remnant are usually used for the treatment of infection, which results in a good prognosis. For patients with urachal carcinoma, resection of the urachus, umbilicus, and partial cystectomy with or without pelvic lymphadenectomy are generally recommended, but the prognosis is limited (18, 19).

Due to the increasing use of abdominal cross-sectional imaging, urachal anomalies are less rare than previously thought, and complications such as malignancy and infection are more commonly detected (16). However, there have been few studied concerning the radiologic features of urachal carcinoma or infection, and even fewer studies focusing on differentiating between the malignancy and infection of the urachus. Nimmonrat A et al. concluded that differentiation between carcinoma and infection is difficult based on imaging alone (20). Therefore, in this study, we describe and compare the clinical and radiological characteristics of urachal carcinoma and infection, explore possible and effective methods to distinguish the two diseases, and ultimately avoid misdiagnosis and ensure optimal management.

## Materials and Methods

### Patient

This retrospective study was approved by the institutional review board of our hospital and the requirement for informed consent was waived. Between February 2012 and November 2020, 27 consecutive patients were included according to the following criteria: 1. underwent surgical or biopsy and pathologically confirmed urachal carcinoma or inflammation; 2. underwent CT or MR examination within 2 weeks before surgery. Of the 13 urachal carcinoma patients, 12 had been examined by enhanced MRI, 6 by both enhanced CT and enhanced MRI, and one by nonenhanced MRI. Of the 14 urachal infection patients, 9 underwent enhanced MRI, and 3 of them also underwent CT scans simultaneously. The remaining 5 were examined by nonenhanced MRI.

### CT Examination Protocol

All the CT examinations were performed using a 64-slice spiral CT scanner (Discovery 750 HD, GE Healthcare). An intravenous contrast agent (iodixanol 320 mg/ml) was injected at the rate of 3.0-3.5 ml/s and then followed by flushing with 20ml saline. Scanning parameters were as follows: tube voltage, 120 kV; automatic tube current; rotation time, 0.6 seconds; detector pitch, 0.984:1; slice thickness: 1.250mm; The arterial phase scan was automatically triggered when the abdominal aorta arrived the enhancement of 120 HU. The portal and delayed phase scans were started 25 seconds and 55 seconds after the arterial phase respectively.

### MR Examination Protocol

All participates were examined at the same 3.0 Tesla MRI scanner (Discovery 750, GE Healthcare, USA) in supine and feet‐first position. The whole pelvic area was covered by a 32-channel torso phased-array coil. Before the examination, the patients were routinely instructed to urinate two hours in advance, then to drink 500-800ml of water, and to refrain from urinating for at least 0.5 hours. Some of the patients underwent DCE-MR (dynamic contrast-enhanced magnetic resonance) examination after the injection of Gd-DTPA.

The pelvic MRI protocol included sagittal, axial T2-weighted imaging, axial T1-weighted imaging, DWI (diffusion-weighted imaging), and DCE-MR imaging. The specific parameters and details are listed in **Table 1**.

**Table 1:** Sequence parameters of the pelvic MRI protocol.

	T1-weighted imaging	T2-weighted imaging	DWI	DCE
Type of sequence	FSE	FRFSE	SE/EPI	LAVA-Flex
Direction	Axial	Axial, sagittal	Axial	Axial
TR (msec)	568	4039	4744	6.2
TE (msec)	6.8	61.7	71.5	3.1
Section thickness (mm)	4	4	4	6
Intersection gap (mm)	1	1	1	3
Field of view (mm)	400 × 400	340 × 340	380 × 380	380 × 304
matrix size	288 × 160	288 × 192	128 × 128	256 × 160
Bandwidth (kHz)	50	62.5	250	125

DWI, diffusion weighted imaging; DCE, dynamic contrast enhanced; FSE, fast spin echo; FRFSE, fast recovery fast spin echo; EPI, echo-planar imaging; LAVA-Flex, liver acquisition with volume acceleration flex; TR, repetition time; TE, echo time; b=0, 600 sec/mm^2^.

### Image Analysis

Two radiologists (X.X.X. and X.X.) with 8 and 16 years of experience in abdominal imaging diagnosis reviewed the CT and MR images respectively through picture archiving and communication system (PACS). Neither observer was aware of the patients’ clinical information, and the differences were resolved through joint discussion until a consensus was reached.

The following features were analyzed for each lesion: size (largest diameter of the lesion), location (dome or anterior wall of the bladder, urachus), shape (round or oval, lobulated or irregular), margin (well or ill-defined), lesion composition (solid, mainly solid, mainly cystic or cystic), calcification (appear or absent), T1 signal intensity (compared to signals from adjacent muscles), T2 signal intensity (compared to signals from adjacent muscles), peripheral lymph nodes (whether there is an increase or enlargement of the peripheral lymph nodes), degree of enhancement (Significant, mild or no enhancement), thickening of adjacent bladder wall (present or absent).

The ADC value was calculated by FireVoxel software (NYU Center for Advanced Imaging Innovation and Research, USA). DWI images were imported into the software, and the ROI was drawn at the largest area of the lesion, and necrosis area and cystic components were excluded. Finally, the ADC map was calculated by the monoexponential model, and the mean ADC value can be obtained.

### Statistical Analysis

All statistical analyses were performed using SPSS (version 24.0, IBM). All tests were two-sided and the *p*-value <0.05 was considered to indicate a statistically significant difference. Quantitative data such as the age and size were expressed as mean ± SD and analyzed by Student’s *t*-test or Mann–Whitney *U* test. Categorical variables including sex, clinical signs and symptoms, location, shape, margin, lesion composition, calcification, T1 signal intensity, T2 signal intensity, peripheral lymph nodes, degree of enhancement, and thickening of adjacent bladder wall were described as counts and compared using the Fisher’s exact test. The sensitivity and specificity of each significant feature were calculated using Clinical Calculator 1 (http://vassarstats.net). Kappa coefficient consistency test was used to evaluate the reliability of the imaging qualitative analysis, and the interobserver agreement was divided into three levels (Kappa ≥ 0.75, good; 0.4 ≤ Kappa < 0.75, moderate; Kappa < 0.4, poor). The intraclass correlation coefficient was used to estimate the measurement consistency of size and ADC value, and the classification was the same as above.

## Results

### Clinical Features

A total of 27 patients with pathologically proven urachal carcinoma or infection were included. The clinical information was summarized in **Table 2**. There were 13 patients (male/female = 12:1; mean age = 45.5 years) in the urachal carcinoma group and 14 patients (male/female = 6:8; mean age = 35.2 years) in the urachal infection group. There was a statistically significant difference between the two groups by sex (*p* = 0.013), but not by age (*p* = 0.076).

**Table 2:** Comparison of clinical characteristics of urachal carcinoma and infection patients.

	Urachal carcinoma(n=13)	Urachal infection(n=14)	*p*
Sex			0.013*
Male	12	6	
Female	1	8	
Age (mean ± SD, year)	45.5 ± 12.7	35.2 ± 16.0	0.076
Clinical signs and symptoms			
Hematuria	11	1	<0.001*
Pollakiuria	1	3	0.596
Dysuria	1	2	1.000
Abdominal pain	0	5	0.041*
Umbilical discharge	0	1	1.000
asymptomatic	2	4	0.648

Data are number of patients. SD, standard deviation. *Significant results.

In the urachal carcinoma group, hematuria (11/13) was the most common complaint, and one of them had pollakiuria and dysuria simultaneously, and 2 cases (2/13) were asymptomatic and accidentally discovered during routine physical examination. In the urachal infection group, clinical manifestation included abdominal pain (5/14), pollakiuria (3/14), dysuria (2/14), hematuria (1/14), and 4 cases had no obvious symptoms.

In the urachal carcinoma group, 12 patients underwent surgical resection including laparoscopic resection of lesions (n = 5), transurethral resection of lesions (n = 1), and robotic laparoscopic of urachal lesions and extended partial cystectomy (n = 6, including 1 case underwent lymph node dissection during surgery and 4 cases underwent postoperative chemotherapy). 1 patient only underwent a needle biopsy and chemotherapy. In the urachal infection group, 11 patients underwent laparoscopic lesion resection, 1 transurethral lesion resection, and 2 robotic laparoscopic of urachal lesions and extended partial cystectomy.

### Imaging Features

**Figure 1:**
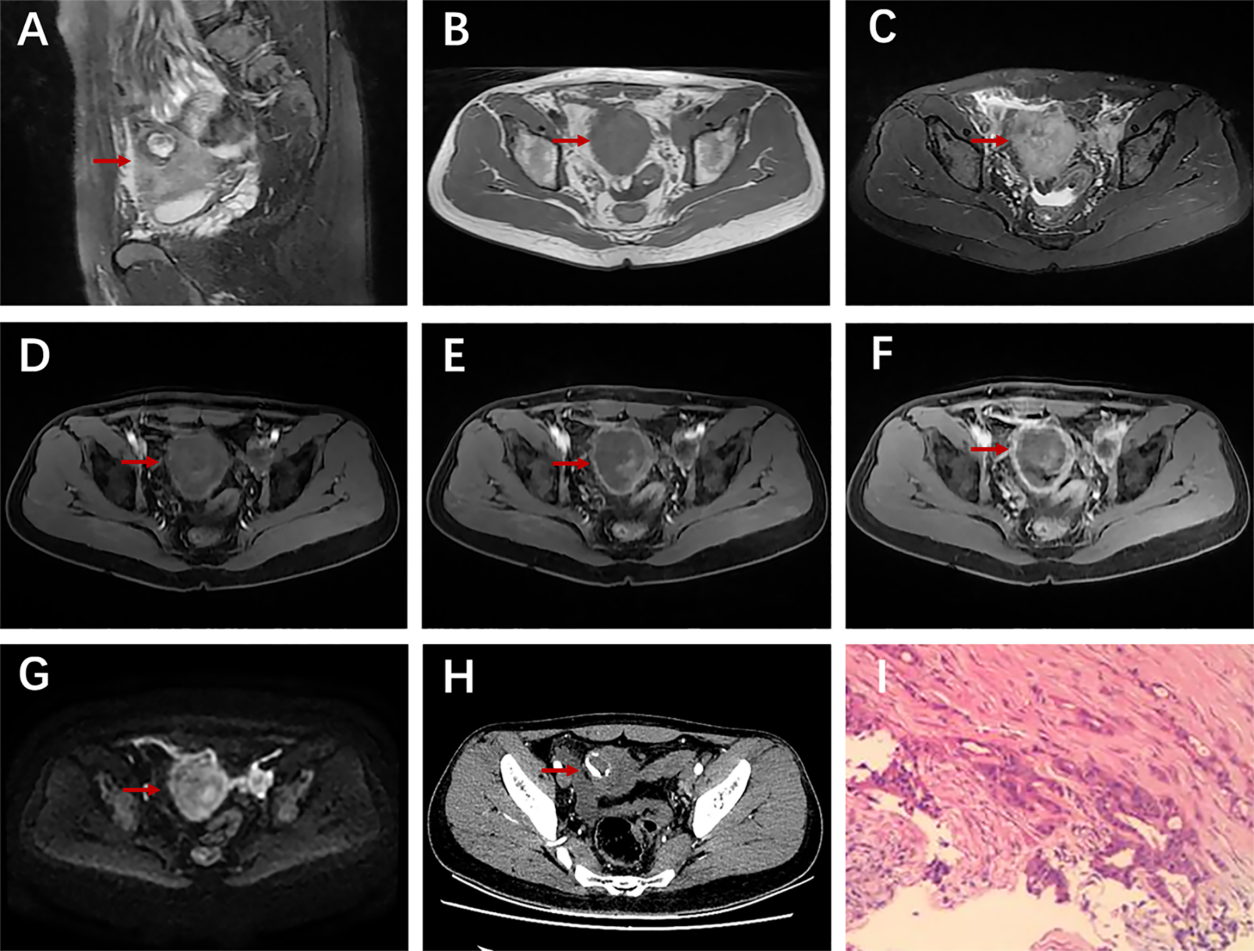
A 28-year-old man with urachal carcinoma presented with hematuria, pollakiuria, and dysuria. Sagittal T2WI **(A)** showed a mass containing cystic elements at the dome of the bladder; iso-intensity signal on T1WI **(B)** and high-intensity signal on T2WI **(C)**; axial dynamic contrast-enhanced LAVA-Flex images showed progressive ring enhancement **(D–F)**; restricted diffusion on DWI especially at the edge of the mass **(G)**; ring-shaped calcification on axial unenhanced CT scan **(H)**; histopathological findings confirmed a moderately differentiated adenocarcinoma of the urachus **(I)**.

All CT and MR imaging characteristics of urachal carcinoma and infections patients are summarized in **Table 3**. Typical cases were showed in **Figures 1** **–** **5**. CT scans of 6 patients with urachal carcinoma showed calcification in 5 of them, whereas none of the 3 patients with urachal infection showed calcification. Thickening of adjacent bladder wall was found in 2 of 13 patients with urachal carcinoma, and 6 of 14 patients with urachal infection. Both features mentioned above were statistically different (*p* = 0.048, *p* = 0.021). However, there was no significant difference in location (*p* = 0.440), shape (*p* = 0.449), margin (*p* = 0.449), lesion composition (*p* = 0.459), T1 signal intensity (*p* = 0.196), T2 signal intensity (*p* = 0.555), peripheral lymph nodes (*p* = 0.236), and degree of enhancement (*p* = 0.184).

**Table 3:** Imaging characteristics of urachal carcinoma and urachal infection.

	Urachal carcinoma (n=13)	Urachal infection(n=14)	*p*	Kappa
Size (mean ± SD, cm)	3.9 ± 1.7	3.7 ± 2.9	0.797	
Location			0.440	1.000
dome or anterior wall of the bladder	6	4		
Urachus	7	10		
Shape			0.449	0.922
Round or oval	7	5		
lobulated or irregular	6	9		
Margin			0.449	0.847
Well-defined	7	5		
Ill-defined	6	9		
Lesion composition			0.459	0.864
Solid	4	3		
Mainly solid	4	4		
Mainly cystic	5	4		
Cystic	0	3		
Calcification			0.048*	1.000
Present	5	0		
Absent	1	3		
Missing	7	11		
T1 Signal intensity			0.196	0.885
Very low	4	1		
Low	4	9		
Iso	4	4		
High	1	0		
T2 Signal intensity			0.555	0.697
Low	0	0		
Iso	1	0		
High	6	9		
Very high	6	5		
Enlarged or increased peripheral lymph nodes			0.236	0.824
Present	6	3		
Absent	7	11		
Degree of enhancement			0.184	0.821
Significant	7	2		
Mild	5	6		
No	0	1		
Missing	1	5		
Adjacent bladder wall thickened			0.021*	0.760
Present	2	6		
Absent	11	8		

Data are number of patients. SD, standard deviation. *Significant results.

**Figure 2:**
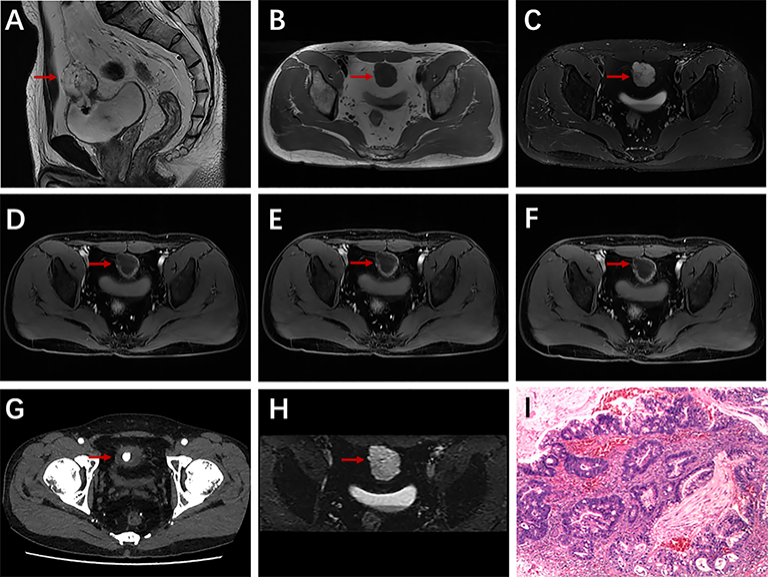
A 43-year-old man with urachal carcinoma presented with painless gross hematuria. Sagittal T2WI **(A)** showed a solid mass in the urachal region; very low signal intensity on T1WI **(B)** and very high signal intensity on T2WI **(C)**; axial dynamic contrast-enhanced LAVA-Flex images showed apparent ring enhancement **(D–F)**; nodular calcification on axial CT scan **(G)**; restricted diffusion on DWI **(H)**; pathologically confirmed urachal adenocarcinoma (intestinal type) **(I)**.

**Figure 3:**
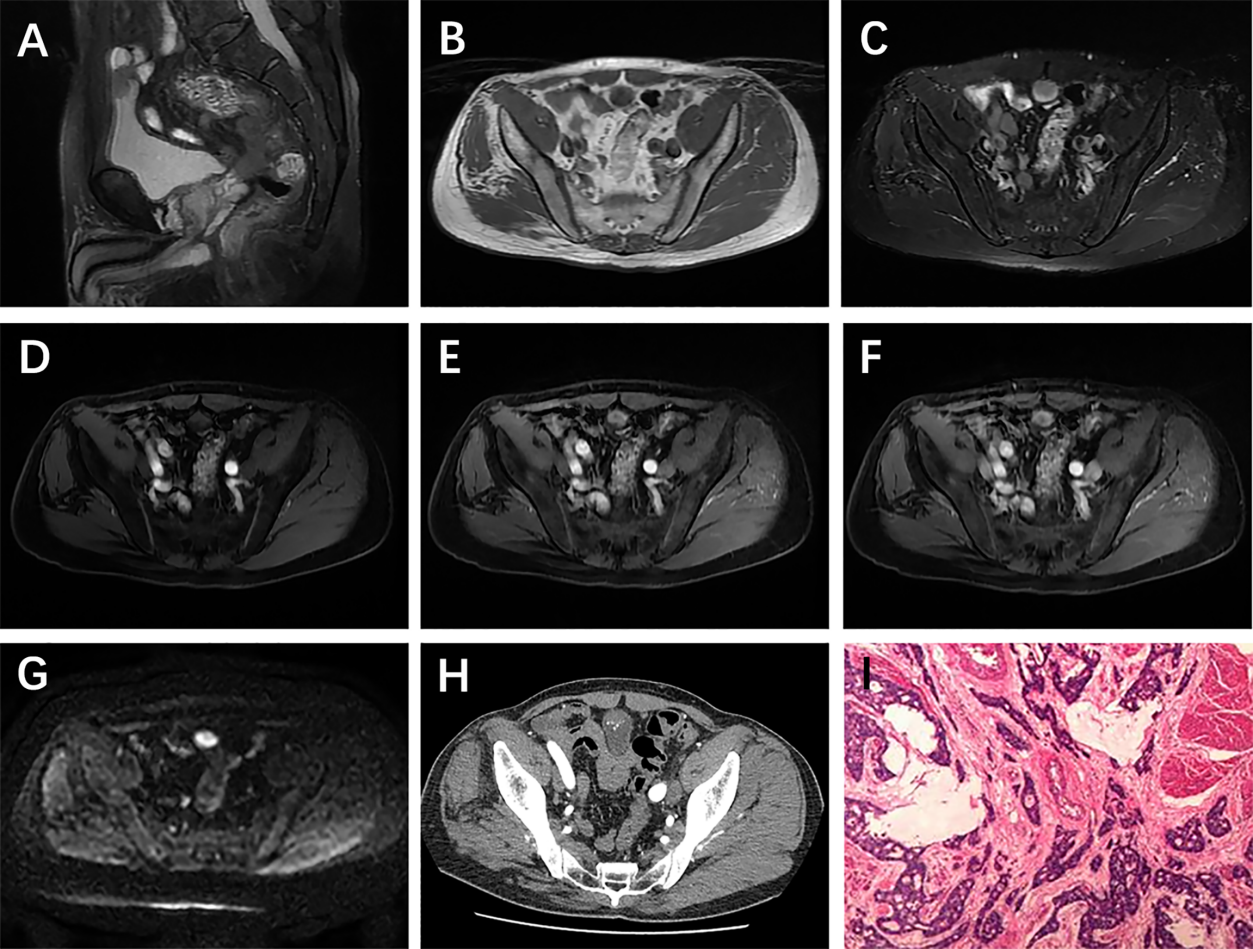
A 62-year-old man with urachal carcinoma presented with painless gross hematuria. Sagittal T2WI **(A)** showed a solid mass in the urachus; slightly low signal intensity on T1WI **(B)** and high signal intensity on T2WI **(C)**; axial dynamic contrast-enhanced LAVA-Flex images showed no obvious enhancement in arterial phase, but obvious enhancement in venous phase and delayed phase **(D–F)**; restricted diffusion on DWI **(G)**; multiple spotted calcifications on axial unenhanced CT scan **(H)**; pathological findings showed moderately differentiated adenocarcinoma (intestinal type) **(I)**.

**Figure 4:**
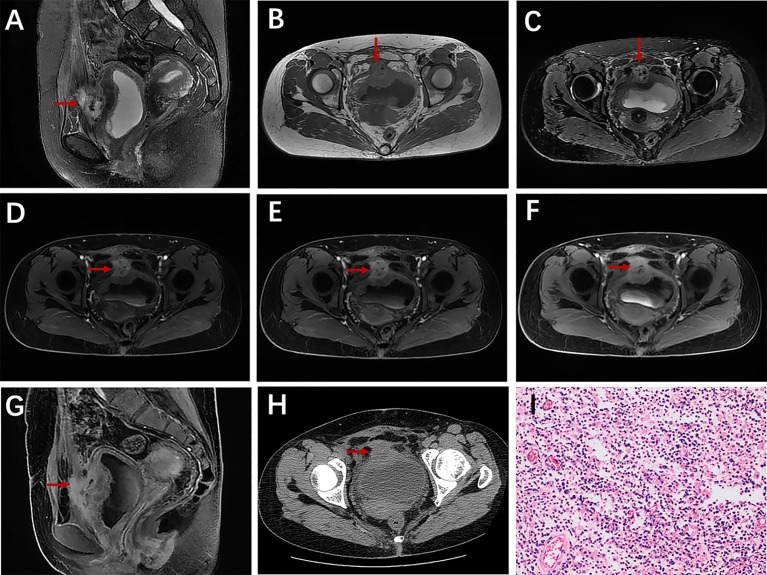
A 38-year-old woman with urachal infection presented with pollakiuria and dysuria. Sagittal T2WI **(A)** showed a mainly solid mass in the urachal region; iso-intensity on T1WI **(B)** and T2WI **(C)**, and the adjacent bladder wall thickened obviously; axial dynamic contrast-enhanced LAVA-Flex images and sagittal contrast-enhanced images **(G)** showed obvious enhancement **(D–F)**; no calcification on axial CT plain scan **(H)**; pathological findings confirmed urachal infection **(I)**.

**Figure 5:**
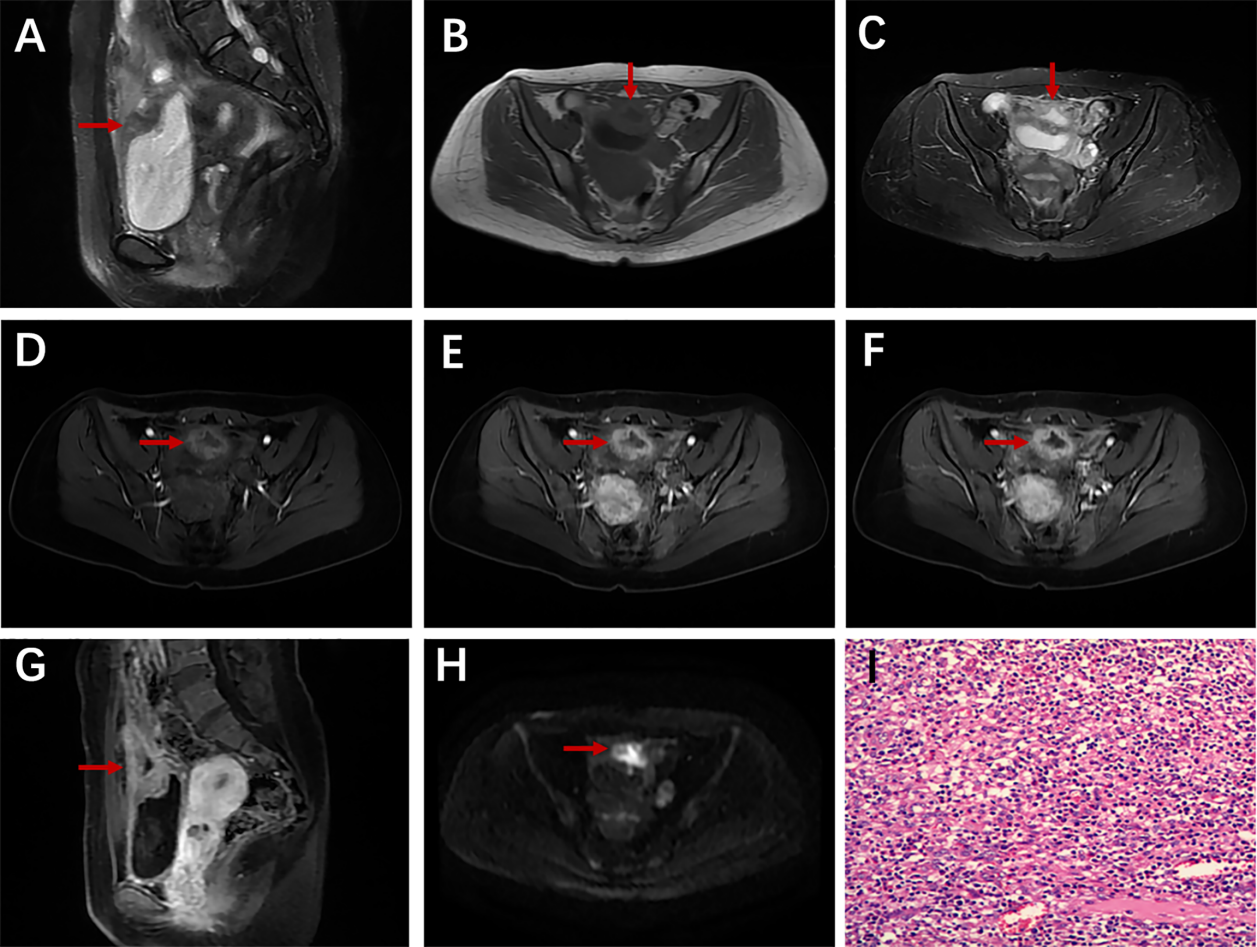
A 32-year-old woman with urachal cyst and infection presented with pollakiuria. Sagittal T2WI **(A)** showed a heterogeneous mass at the junction of the urachus and bladder; the cyst wall showed iso-intensity on T1WI **(B)** and slightly hyperintensity on T2WI **(C)**; axial dynamic contrast-enhanced LAVA-Flex images showed apparent enhancement of the cyst wall **(D–G)**; mildly restricted diffusion in the cyst wall on DWI **(H)**; Pathology showed a large number of acute and chronic inflammatory cell infiltration **(I)**.

6 patients in the urachal carcinoma group underwent DWI examination totally, with the ADC values of 1.280 × 10^-3^ mm^2^/s, 1.820 × 10^-3^ mm^2^/s, 2.115 × 10^-3^ mm^2^/s, 1.536 × 10^-3^ mm^2^/s, 1.381 × 10^-3^ mm^2^/s, 1.630 × 10^-3^ mm^2^/s, and an average of 1.627 × 10^-3^ mm^2^/s. 7 patients in the urachal infection group were also examined by DWI, the ADC values were 1.156 × 10^-3^ mm^2^/s, 1.965 × 10^-3^ mm^2^/s, 1.330 × 10^-3^ mm^2^/s, 1.526 × 10^-3^ mm^2^/s, 1.415 × 10^-3^ mm^2^/s, 1.913 × 10^-3^ mm^2^/s, 2.538 × 10^-3^ mm^2^/s, and the average ADC value was 1.692 × 10^-3^ mm^2^/s. There was no significant difference in ADC values between urachal carcinoma and infection.

#### Pathologic Findings

There were 13 patients pathologically confirmed as urachal adeno-carcinoma, including 1 case of highly differentiated adenocarcinoma, 6 cases of moderately differentiated adeno-carcinoma, 1 case of moderate to low differentiated adenocarcinoma, 2 cases of mucous adenocarcinoma, 1 case of signet-ring carcinoma, and 2 cases of intestinal-type adenocarcinoma. The tumors were staged according to the Mayo staging system (**Table 4**). There were 11 patients in stage II and 2 in stage IV. The remaining 14 cases were confirmed as inflammatory cell infiltration, of which 7 cases were secondary to the urachal cyst, 1 case secondary to urachal sinus, and 6 cases secondary to patent urachus.

**Table 4:** The Mayo staging system of urachal cancer.

Stage	Mayo classification
I	tumors confined to the urachus/bladder
II	tumors extending beyond the muscular layer of the urachus/bladder
III	tumors infiltrating the regional lymph nodes
IV	tumors infiltrating non-regional lymph nodes/distant metastases

#### Sensitivity and Specificity of Significant Features for the Diagnosis

The sensitivity, specificity and 95% CI (confidence interval) of significant features for distinguishing between urachal carcinoma and urachal infection are showed in **Table 5**. Among them, the sensitivity and specificity of hematuria and calcification in distinguishing urachal carcinoma from infection were both more than 80%.

**Table 5:** Sensitivity and specificity of significant parameters.

Criteria	Sensitivity (%)	95% CI (%)	Specificity (%)	95% CI
Female	57.14 (8/14)	29.65-81.19	92.31 (12/13)	62.09-99.60
Hematuria	84.62 (11/13)	53.66-97.29	92.85 (13/14)	64.17-99.63
Abdominal pain	38.46 (5/13)	15.13-67.72	1 (14/14)	73.24-1
Calcification	83.33 (5/6)	36.48-99.12	1 (3/3)	31.00-1
Thickening of adjacent bladder wall	42.86 (6/14)	18.81-70.35	84.62 (11/13)	53.66-97.29

CI, confidence interval; Data in parentheses are number of lesions.

### Interobserver Agreement

For qualitative characteristics except for T2 signal intensity (Kappa = 0.697), the Kappa values were all greater than 0.75 (Kappa = 0.760-1.000), indicating good consistency. For quantitative features including the lesion size and ADC value, both interobserver agreements were very good (ICC = 0.997 and 0.916, respectively).

## Discussion

In this study, we have two main findings. Clinically, hematuria has the most distinguishing significance. In imaging, calcification is an important feature of urachal carcinoma, and infection usually manifests as thickening of the adjacent bladder wall. In summary, clinical and imaging features may be helpful for the differentiation between urachal carcinoma and infection.

Clinical signs and symptoms are variable, usually non-specific, and accidentally discovered (21). Consistent with the previous studies (10, 14, 22, 23), hematuria (11/13) was the most common symptom of urachal carcinoma in our study, but it was rare in urachal infections, and the difference was statistically significant. This may be mainly because the tumor may invade and ulcerate the bladder mucosa (14). According to previous studies (24–26), hematuria is usually the chief complaint of urachal carcinoma, so we believe that hematuria may be one of the most important indications for the diagnosis of urachal carcinoma.

We also found that abdominal pain between these two types of diseases is statistically different. A review article concluded that 14 percent of patients with urachal carcinoma may experience abdominal pain (10). Previous studies have also reported that infected urachus may manifest as abdominal pain (27–29). Based on our data, abdominal pain was more common in urachal infections. In this study, only one patient in the urachal infection group showed umbilical discharge, and this infection was secondary to patent urachus. Because both the patent urachus and umbilical-urachal sinus are connected to the umbilical cord, it is easy to understand that these two infections may be accompanied by a purulent discharge (15). And umbilical discharge may be the specific clinical presentation of urachal infection. Mucosuria is a characteristic symptom and may raise the possibility of urachal malignancy (22, 30), but it is relatively unusual and was not observed in the current study. In addition, we also found that urachal carcinoma was more common in men (male: female = 12:1), while the infection showed no gender predominance (male: female = 6: 8).

Imaging findings may also be used to diagnose and distinguish urachal lesions. According to some previous studies, calcification is a characteristic sign of urachal carcinoma (8, 10, 22). Approximately 50% to 70% of urachal carcinoma may present punctate, speckled, nodular, or curvilinear calcification in or around the center of the tumor (31). However, Carolina Parada Villavicencio et al. (16) point out that due to chronic urine retention, especially in patients with a urachal cyst or vesicourachal diverticula, calcification can also occur in urachal infection. In this study, the majority of patients with urachal carcinoma (5/6) showed calcification on CT imaging, while none of the patients (0/3) with urachal infection showed calcification. Although the sample size was small, the difference was statistically significant, and we could also assume that calcification was more common in urachal carcinoma.

With the development of MR imaging technology, combined with axial and sagittal images, the involvement of adjacent structures could be determined (16, 20). Nimmonrat A and colleagues (20) reported that both urachal carcinoma and infection may exist in the form of solid mass and involve adjacent organs. In our study, urachal carcinoma may present as solid, mainly solid, mainly cystic rather than cystic form, and urachal infection may present as one of the four components mentioned above. Simple cystic lesions may be more likely to be infections, but we still cannot distinguish between the two by the components of the lesion. Based on multiplanar MR images, only 2 of 13 patients with urachal carcinoma showed thickening of the adjacent bladder wall, while 6 of 14 patients with urachal infection showed it. The difference was statistically significant, indicating whether the infection is more likely to invade the surrounding bladder wall, which requires further research to confirm.

As for other imaging features, the differences were not statistically significant. Both urachal carcinoma or infection could be located in the dome or anterior of the bladder or urachus, with a regular or irregular shape, well-defined or ill-defined margins, low or equal T1 signal, high T2 signal, varying degrees of enhancement, and with or without peripheral lymph node enlargement or increase. The study of Nimmonrat A et al. (20) concluded that it is very difficult to distinguish urachal carcinoma from infection based on ultrasonography (US) and CT imaging. In our study, combined with clinical features, mainly based on CT and MR, especially MR, the high resolution of soft tissue can provide much more imaging information (16).

There were several limitations in this study. First, our study was retrospective and the selection and verification bias could not be avoided. Second, the sample size of our study was relatively small, in particular, CT examination was performed for only 6 cases of urachal carcinoma and 3 cases of urachal infection. Therefore, the reliability of the statistical results was restricted. Finally, there are few studies related to urachal carcinoma or infection, and the literature we can refer to is very limited, which may lead to some limitations to a certain extent.

## Conclusion

In conclusion, our data show that for the identification of urachal lesions, hematuria, and calcification are more likely to be urachal carcinoma, while female, abdominal pain, and thickening of adjacent bladder wall are more likely to be infections. Combining clinical and radiological features can help distinguish urachal carcinoma from urachal infection.

## Data Availability Statement

The original contributions presented in the study are included in the article/supplementary material. Further inquiries can be directed to the corresponding author.

## Author Contributions

Study concepts: ZL. Study design: ZL and XM. Data acquisition: SL and PL. Data analysis and interpretation: XM and YS. Statistical analysis: SL. Manuscript preparation and editing: SL and XM. Manuscript review: ZL and DH. All authors contributed to the article and approved the submitted version.

## Funding

This study is supported by the grants from National Natural Science Foundation of China (NSFC) No. 82071889, 81771801, and 82071890.

## Conflict of Interest

The authors declare that the research was conducted in the absence of any commercial or financial relationships that could be construed as a potential conflict of interest.

## Publisher’s Note

All claims expressed in this article are solely those of the authors and do not necessarily represent those of their affiliated organizations, or those of the publisher, the editors and the reviewers. Any product that may be evaluated in this article, or claim that may be made by its manufacturer, is not guaranteed or endorsed by the publisher.
